# Preventing bleeding and surgical complications in transoral robotic surgery for the oropharynx: results of a global survey

**DOI:** 10.3389/fonc.2026.1864700

**Published:** 2026-07-20

**Authors:** Elena Russo, Gian Marco Pace, Andrea Costantino, Giannicola Iannella, Claudio Sampieri, Armando De Virgilio

**Affiliations:** 1 Department of Sense Organs, ‘Sapienza’ University of Rome, Rome, Italy; 2 Division of Reconstructive Microsurgery, Department of Plastic and Reconstructive Surgery, Chang Gung Memorial Hospital, Taoyuan, Taiwan; 3 Department of Biomedical Sciences, Humanitas University, Milan, Italy; 4 Otorhinolaryngology Unit, Istituto di ricovero e cura a carattere scientifico (IRCCS) Humanitas Research Hospital, Milan, Italy; 5 Unit of Head and Neck Tumors, Hospital Clínic, Barcelona, Spain

**Keywords:** bleeding, head and neck, oropharynx, surgical complication, transoral robotic surgery

## Abstract

**Objective:**

This study aimed to characterize global practice patterns in transoral robotic surgery (TORS), with particular attention to differences across oropharyngeal subsites.

**Methods:**

We conducted a 40-item international web-based survey of TORS-experienced head and neck surgeons (April–June 2024). The questionnaire covered demographics, robotic platforms, bleeding prevention, airway management, postoperative care, and reconstruction. Descriptive statistics summarized responses. Base of tongue resections were compared with lateral oropharyngectomies using χ² or Fisher’s exact tests, with Cramér’s V for effect sizes; McNemar’s test was used for paired binary data.

**Results:**

Sixty-nine surgeons from 22 countries completed the survey (response rate 37.9%), most working in academic centers (73.9%). Prophylactic neck vessel ligation was routinely performed by about 60% of surgeons for both base of tongue and lateral oropharyngectomy, with no significant difference in overall use between subsites. Among surgeons performing ligation, lingual artery ligation was significantly more frequent in base of tongue procedures than in lateral oropharyngectomy (p < 0.001; Cramér’s V = 0.46). Routine tracheotomy was uncommon (4–9%) and mainly reserved for selected high-risk cases. Postoperative care varied substantially, with most patients hospitalized for ≥4 days and nasogastric tube use in 58–64% of cases. Local flap reconstruction was significantly more common after lateral oropharyngectomy than base of tongue resection, whereas free flap use did not differ between subsites.

**Conclusion:**

Our findings highlight considerable variability in perioperative and intraoperative management strategies for oropharyngeal TORS, reflecting the lack of standardized protocols. Differences in bleeding prevention and reconstructive techniques may be related to the anatomy of specific oropharyngeal subsites, underscoring the need for consensus-building efforts to harmonize TORS patient management.

## Introduction

Approved by the FDA in 2009 for head and neck procedures, transoral robotic surgery (TORS) has since gained widespread acceptance and use due to its precision, reduced patient morbidity, and faster recovery times (1). Since its approval, it has spread worldwide, carving out an increasingly central role in the surgery of the upper aerodigestive tract. In particular, in oropharyngeal surgery, transoral robotic surgical techniques have enabled minimally invasive access and significantly reduced the need for open surgical approaches in many oropharyngeal surgery cases. Traditional approaches tend to be more invasive and can result in more significant functional sequelae, making TORS a potentially more advantageous option for both surgeons and patients (1). Beyond these advantages, TORS can offer a superior visualization of the surgical field, enhanced dexterity with robotic instruments, and a reduction in operative times. However, robotic surgery carries its own set of risks and potential complications, and bleeding is one of the most feared (2). Therefore, careful intraoperative and perioperative management of the patient is essential to mitigate these risks, as effective management strategies are just as crucial as the surgical procedure itself in ensuring a successful outcome.

As a relatively new technique, still limited to selected centers and given the variety of robot models used in head and neck surgery to approach the oropharynx, there is still no consensus on how to avoid intraoperative and perioperative complications (3). Patient care protocols vary significantly from country to country, hospital to hospital, and even surgeon to surgeon.

In this survey, we aimed to analyze the routine practices of TORS surgeons worldwide, focusing on the different subsites of the oropharynx. We examined the instruments used, strategies to prevent the risk of bleeding, airway protection methods, and patient care pathways following TORS procedures. Our goal was to capture diverse perspectives and highlight variations among institutions and surgeons, challenging preconceived notions based on individual centers. By providing an overview of prevailing trends and areas of variation in clinical practice, we aimed to offer a comprehensive analysis of current TORS management strategies.

## Methods

### Survey design and distribution

A 40-item survey was developed by a head and neck surgeon with over 10 years of experience in TORS. Before distribution, the questionnaire was pilot-tested with a small group of head and neck surgeons from different institutions to assess clarity, relevance, and comprehensibility. Feedback from this process was used to refine the wording, improve the overall structure, and ensure consistency in the interpretation of survey items. The survey was administered exclusively in English; therefore, no translation or cross-cultural adaptation process was required. The final survey was conducted between April 29, 2024, and June 8, 2024. Surgeons were identified through a targeted literature search of authors who had published on TORS, complemented by the professional networks of the study team across multiple regions, including Europe, the United States and Asia. This method produced a convenient sample of clinicians with proven previous experience in performing TORS. The email invitation included a brief description of the study’s aims and a direct link to the survey, and a reminder email was sent two weeks later to encourage participation. Informed consent was implied by the completion of the survey. Participation was entirely voluntary, and all responses were anonymized to ensure participant confidentiality. Ethical approval was not required, as the study did not involve patient data or any identifiable institutional information.

### Survey content

A web-based survey was created using Google Forms (Mountain View, CA, USA) and included both closed- and open-ended questions to gather quantitative and qualitative information. The survey addressed four main areas related to TORS: respondent demographics and professional background (including name, affiliation, country, hospital type, years of experience as both primary surgeon and assistant, and main robotic platform used); clinical practice patterns and procedural preferences (such as most frequently performed TORS procedures and routinely used instruments for different anatomical subsites); perioperative management strategies (covering practices on cervical vessel ligation, indications and duration of tracheotomy, use and duration of nasogastric tube, length of hospitalization, and timing to resume a full oral diet); and reconstructive and hemostatic approaches (use of local and free flaps, application of hemostatic agents, and specific products used). Open-ended responses were coded and analyzed by two experienced qualitative researchers, and themes were identified. The complete survey questionnaire can be found in the Supplementary Material (**Appendix A**). The survey was configured such that all questions were mandatory, preventing submission of incomplete questionnaires. To avoid duplicate entries, only one response per e-mail address was permitted. Respondents were able to review and modify their answers before submitting the survey; however, no changes could be made after submission. The answers were self-reported by participants relying on their clinical experience and were not validated against institutional or surgical databases. Because the questionnaire did not set a specific reporting timeframe (for example, lifetime or recent activity), the responses likely reflect the sum of each participant’s career experience.

### Data analysis

The data were summarized using descriptive statistics. Continuous variables were presented as medians and interquartile ranges (IQRs). Categorical variables were reported as counts and percentages. For multi-response questions, percentages were calculated using the number of respondents eligible for the specific response category as the denominator. The chi-squared test (χ²) was used to ascertain the level of significance when comparing different oropharyngeal subsites. Multi-response questions were dichotomized into no/yes groups based on the presence of one or more affirmative responses to ensure the mutually exclusive assumption of the χ² test was not violated. Fisher’s exact test was used when expected cell counts were <5. Effect sizes were calculated using Cramér’s V. Given the paired nature of the data, McNemar’s test was additionally performed as a sensitivity analysis for binary outcomes. All statistical tests were performed using R version 4.4.0. Statistical significance was defined as p < 0.05.

## Results

### Participants

**Figure 1:**
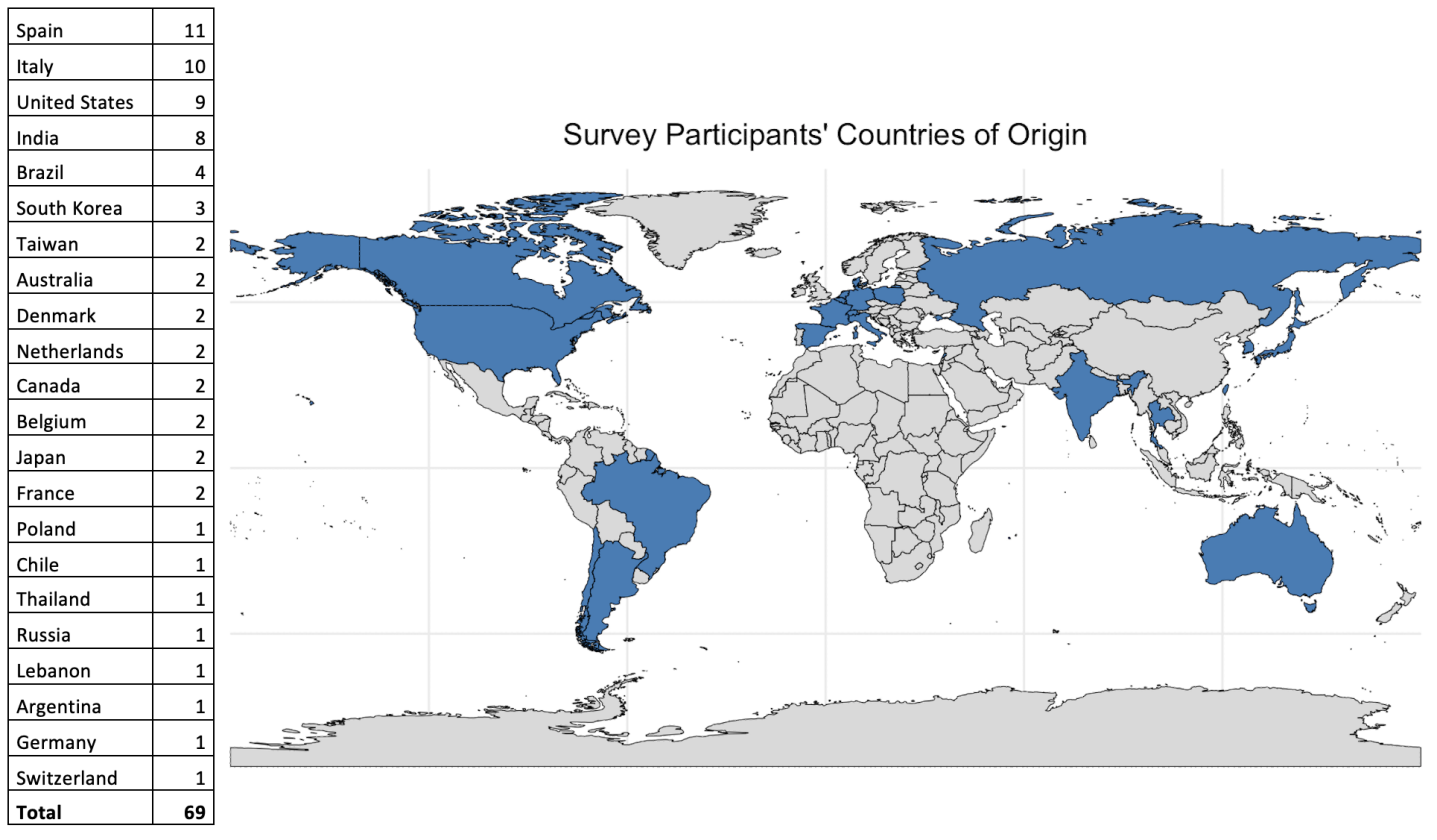
Survey participants’ countries of origin.

The demographic data of the survey participants are presented in **Table 1**. Among the 182 head and neck surgeons who received the email, a total of 69 participated in the survey, resulting in a 37.9% response rate. Most of the surgeons were males (N = 63/69, 91.3%), and affiliated to academic hospitals (N = 51/69, 73.9%). The median age was 49 (IQR 42 - 57). Survey respondents worked in hospitals across 22 different countries, with 11 participants from Spain (15.9%), 10 from Italy (14.5%), 9 from the United States (13.0%), and 8 from India (11.6%) (**Figure 1**). The majority of the participants (N = 44/69, 63.7%) had at least 5 years of experience in performing TORS. Most of the surgeons (N = 46/69, 66.6%) perform at least 10 TORS procedures per year as console surgeons, while it is less common to be bedside assistants, with 51 (73.9%) surgeons assisting less than 10 TORS every year. The da Vinci Xi is the most frequent robot used (N = 47/69, 68.1%), followed by the da Vinci Single Port (N = 13/69, 18.8%). Monopolar spatula (N = 59/69, 85.5%) and Maryland bipolar (N = 58/69, 84%) are the most frequently used instrument regardless of the site of excision, followed by monopolar curved scissors (N = 16/69, 23.2%), grasp forceps (N = 13/69, 18.8%), and fenestrated bipolar forceps (N = 10/69, 14.5%).

**Table 1:** Survey participants’ demographic data. IQR, interquartile range.

Age	49 (IQR 42 - 57)
Sex	
Female	6 (8.7%)
Male	63 (91.3%)
Type of medical center:	
Non-University	18 (26.1%)
University	51 (73.9%)
Experience in transoral robotic surgery (years):	
< 1	2 (2.9%)
> 10	23 (33.3%)
1 - 2	10 (14.5%)
2 - 5	13 (18.8%)
5 - 10	21 (30.4%)
Average number of procedures per year as a main surgeon:	
> 50	6 (8.7%)
0 - 4	5 (7.2%)
11 - 20	15 (21.7%)
21 - 30	13 (18.8%)
31 - 40	8 (11.6%)
41 - 50	4 (5.8%)
5 - 10	18 (26.1%)
Average number of procedures per year as an assistant:	
> 50	3 (4.3%)
0 - 4	32 (46.4%)
11 - 20	6 (8.7%)
21 - 30	7 (10.1%)
31 - 40	2 (2.9%)
5 - 10	19 (27.5%)
Robotic system mainly used:	
Da Vinci Si	8 (11.6%)
Da Vinci SP	13 (18.8%)
Da Vinci Xi	47 (68.1%)
Xi and SP	1 (1.4%)

### Bleeding prevention

**Figure 2:**
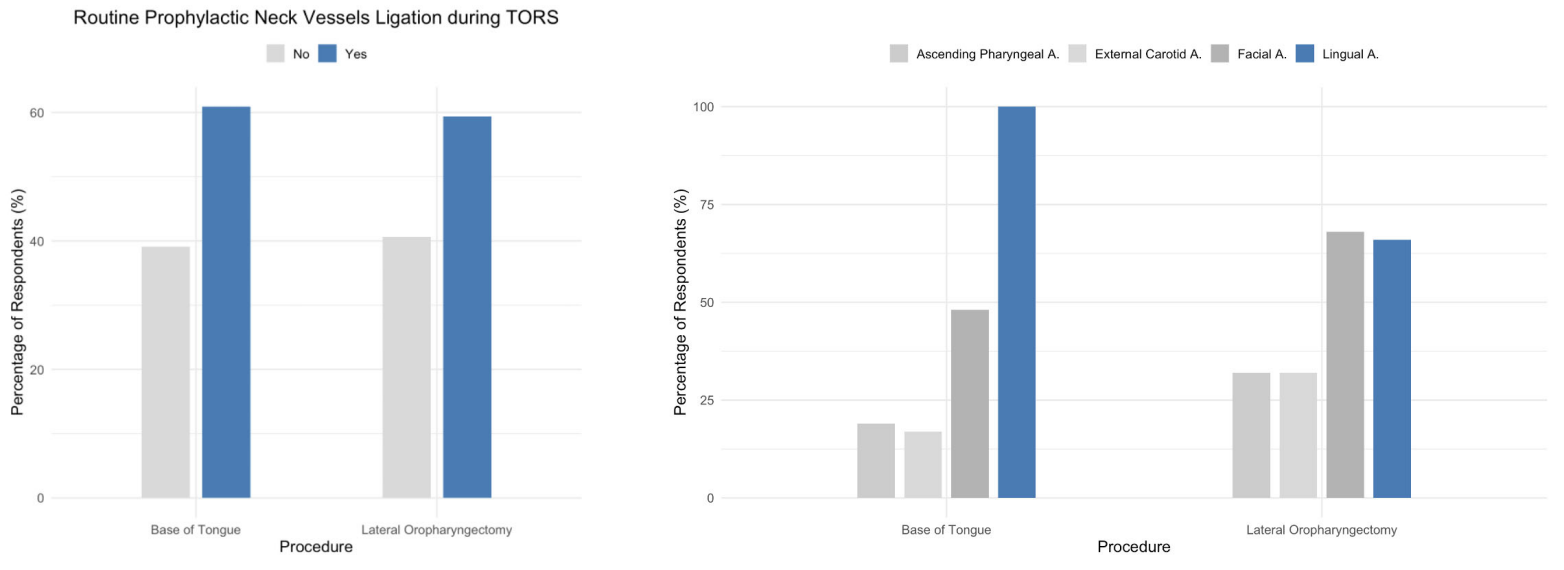
Routine prophylactic neck vessels ligation during TORS.

Prophylactic neck vessels ligation is routinely performed for base of tongue procedures by 60.9% (N = 42/69) of surgeons. The lingual artery is ligated in all cases (100%, N = 42/42), followed by the facial (48%, N = 20/42), ascending pharyngeal (19%, N = 8/42), and external carotid (17%, N = 7/42) arteries (**Figure 2**).

For lateral oropharyngectomy, 59.4% (N = 41/69) of surgeons routinely perform prophylactic neck vessels ligation. The facial (68%, N = 28/41) and lingual (66%, N = 27/41) arteries are the most frequently ligated, followed by the ascending pharyngeal (32%, N = 13/41), and external carotid (32%, N = 13/41) arteries (**Figure 2**).

No significant difference was observed between the two subsites in the routine use of prophylactic neck vessel ligation (p = 0.86, **Table 2**). However, among surgeons who routinely ligate neck vessels, lingual artery ligation was performed significantly more frequently in base of tongue procedures compared with lateral oropharyngectomy (p < 0.001, **Table 2**), with a large effect size (Cramér’s V = 0.46, **Table 2**).

**Table 2:** Comparison of perioperative management, airway strategy, postoperative course, and reconstructive choices between base of tongue and lateral oropharyngectomy procedures.

Variable	BOT (%)	Lateral oropharyngectomy (%)	Test	p-value	Cramér’s V
Bleeding prevention					
Prophylactic neck vessel ligation	42 (60.9)	41 (59.4)	χ²	0.86	0.02
Hemostatic agent use	29 (42.0)	33 (47.8)	χ²	0.47	0.07
Lingual artery ligation^†^	42 (100)	27 (65.9)	χ²	<0.001	0.46
Airway management					
Routine tracheotomy	6 (8.7)	3 (4.3)	Fisher	0.49	0.11
Postoperative course					
Hospitalization time ≥ 4 days	53 (76.8)	49 (71.0)	χ²	0.78	0.11
Nasogastric tube use	44 (63.8)	40 (58.0)	χ²	0.46	0.07
Time to return to full oral diet	13 (18.8)	19 (27.5)	χ²	0.497	0.16
Reconstruction					
Local flap reconstruction	29 (42.0)	43 (62.3)	χ²	0.017	0.20
Local flap for carotid exposure ^§^	14 (20.3)	31 (44.9)	χ²	0.002	0.26
Local flap for major vessel or carotid exposure ^§^	20 (29.0)	36 (52.2)	χ²	0.006	0.24
Free flap reconstruction	39 (56.5)	44 (63.8)	χ²	0.28	0.12

^†^ Percentages calculated among surgeons routinely performing prophylactic neck vessel ligation.

^§^ Subgroup analyses.

Values are expressed as numbers (percentages). Comparisons were performed using χ² or Fisher’s exact test, with effect size reported as Cramér’s V. Subgroup analyses are specified in the table. BOT, base of tongue.

Finally, hemostatic agents are routinely used to prevent bleeding at the end of base of tongue and lateral oropharyngectomy procedures by 42.0% (N = 29/69) and 47.8% (N = 33/69) of surgeons, respectively, with no significant difference between subsites (p = 0.47, **Table 2**).

Sensitivity analysis using McNemar’s test for paired binary data confirmed the χ² findings (**Supplementary Table 1**).

### Tracheotomy

**Figure 3:**
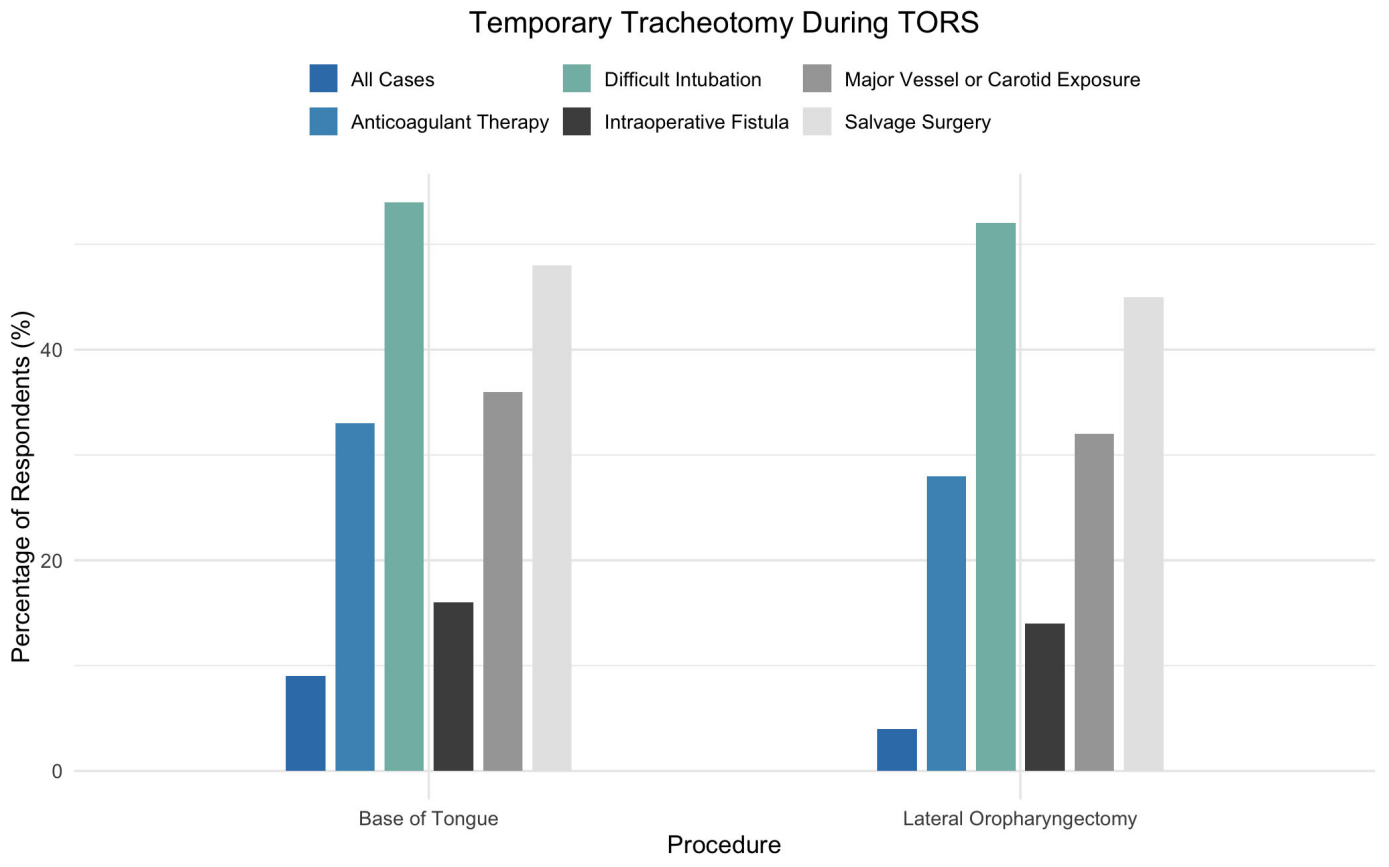
Temporary tracheotomy during TORS.

A temporary tracheotomy is rarely performed routinely for base of tongue procedures (9%, N = 6/69). The most common indications are difficult intubation (54%, N = 37/69), salvage surgery (48%, N = 33/69), major vessel or carotid exposure (36%, N = 25/69), anticoagulant therapy (33%, N = 23/69), and intraoperative fistula (16%, N = 11/69) (**Figure 3**).

Similarly, a temporary tracheotomy is rarely performed for lateral oropharyngectomies (4%, N = 3/69). The most common indications are difficult intubation (52%, N = 36/69), salvage surgery (45%, N = 31/69), major vessel or carotid exposure (32%, N = 22/69), anticoagulant therapy (28%, N = 19/69), and intraoperative fistula (14%, N = 10/69) (**Figure 3**).

No statistically significant difference in routine tracheotomy rates was found between subsites (p = 0.49, **Table 2**), with concordant results on McNemar’s test (**Supplementary Table 1**).

### Postoperative course

**Figure 4:**
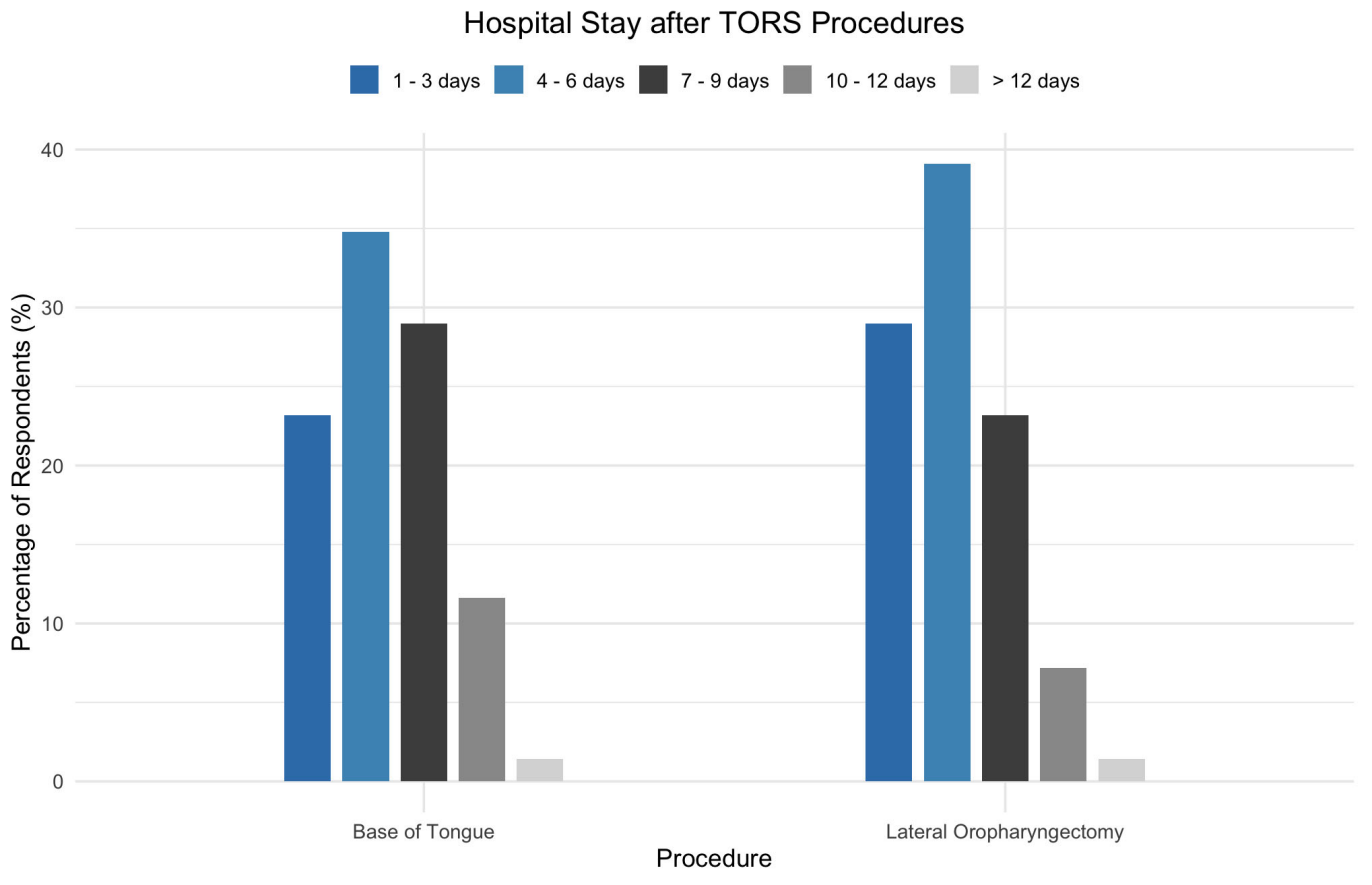
Hospital stay after TORS.

Significant variability in hospitalization time was observed among surgeons. The majority of patients remained hospitalized for at least 4 days after base of tongue procedures (76.8%, N = 53/69) and lateral oropharyngectomies (71.0%, N = 49/69) (**Figure 4**), with no significant difference between subsites (p = 0.78, **Table 2**).

**Figure 5:**
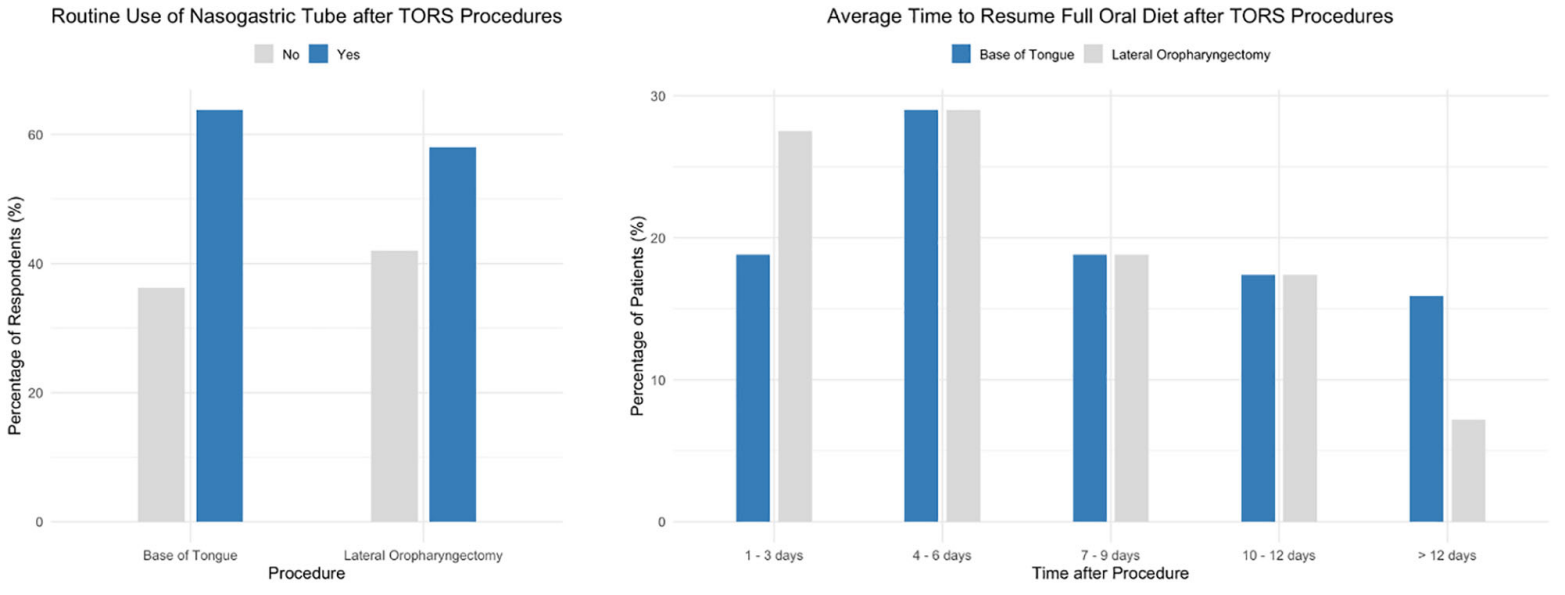
Nasogastric tube positioning and resumption of full oral diet.

A nasogastric feeding tube is preventively used in 64% (N = 44/69) and 58% (N = 40/69) of cases of base of tongue resections and lateral oropharyngectomies, respectively, without a statistically significant difference (p = 0.46, **Table 2**). An evident variability was measured among surgeons regarding the time to full oral diet resumption. Only a minority of surgeons aim to achieve a full oral diet in the first 3 post-operative days for base of tongue procedures (18.8%, N = 13/69) and lateral oropharyngectomies (27.5%, N = 19/69) (**Figure 5**), with no significant difference between subsites (p = 0.50, **Table 2**).

McNemar’s test for paired data confirmed the absence of significant differences between subsites for postoperative course variables (**Supplementary Table 1**).

### Flap reconstruction

Regarding flap reconstruction, 20 participants (29.0%) reported that they never perform a flap after TORS.

Local flap reconstruction was performed significantly more frequently after lateral oropharyngectomy than after base of tongue resection (62.3%, N = 43/69 vs 42.0%, N = 29/69; p = 0.017, **Table 2**). This difference remained significant on McNemar’s test for paired data (**Supplementary Table 1**) and was associated with a moderate effect size (Cramér’s V = 0.20, **Table 2**). The difference was primarily driven by cases with carotid and other major vessel exposure. Local flap use for carotid exposure was significantly higher in lateral oropharyngectomy compared with base of tongue resection (p = 0.002; Cramér’s V = 0.26), as was local flap use for major vessel or carotid exposure (p = 0.006; Cramér’s V = 0.24; **Table 2**).

The most common indications for local flaps after base of tongue resections are major vessel or carotid exposure (69%, N = 20/29), intraoperative fistula (55%, N = 16/29), and salvage surgery (41%, N = 12/29). A similar trend was observed for performing local flaps in lateral oropharyngectomies, with major vessel or carotid exposure (84%, N = 36/43), intraoperative fistula (58%, N = 25/43), and salvage surgery (42%, N = 18/43) noted.

Free flap reconstruction was used after lateral oropharyngectomies in 63.8% of cases (N = 44/69), and after base of tongue resections in 56.5% of cases (N = 39/69), with no statistically significant difference between subsites (p = 0.28, **Table 2**). McNemar’s test for paired data confirmed this finding (**Supplementary Table 1**). The most common indications for free flaps after base of tongue resections are salvage surgery (69%, N = 27/39), major vessel or carotid exposure (67%, N = 26/39), and intraoperative fistula (62%, N = 24/39). A similar trend for performing free flaps was found for lateral oropharyngectomies: major vessel or carotid exposure (70%, N = 31/44), salvage surgery (70%, N = 31/44), and intraoperative fistula (50%, N = 22/44).

## Discussion

Over the past two decades, the number of TORS procedures has significantly increased in head and neck surgery, underscoring its growing significance in this field. The remarkable oncologic and functional outcomes, along with decreased hospitalization lengths and complication rates, have also contributed to an improved quality of life for patients (4, 5). However, despite its numerous benefits, the adoption of robotic systems in otorhinolaryngology still lags behind other specialties, such as urology and gynecology (6). Perioperative and intraoperative strategies to prevent surgical complications, especially postoperative bleeding, lack standardization and often rely on the specific practices and experience of each center and surgeon. This survey was conducted to evaluate the standard practices of TORS surgeons globally. We focused on strategies for reducing bleeding risks, methods for airway protection, and postoperative care strategies after TORS procedures, aiming to describe current practice patterns, identify areas of variability, and highlight aspects of perioperative management that lack standardization.

In this survey, 73.9% of respondents practiced in academic hospital settings, likely reflecting both the concentration of TORS expertise in tertiary referral centers and our recruitment strategy, which relied on surgeons identified through the published TORS literature and professional networks. Accordingly, while the following findings provide valuable insight into contemporary practice among experienced TORS surgeons, they may not fully represent practices in lower-volume or non-academic settings. Therefore, caution is warranted when generalizing these observations more broadly.

In recent years, the Single Port (SP) Da Vinci system has introduced significant innovations, offering a safety profile comparable to multiport systems (Si/Xi) and advantages like reduced docking and console times due to improved maneuverability (7). Despite these advancements, over 68% of surgeons still utilize the Da Vinci Xi system, and only 17% have switched to the SP system. To note, the adoption of a specific robotic system is extremely dependent on the healthcare system, with North America and South Korea being the first in the use of the SP system, which was only recently approved in Europe. Finally, the Monopolar Spatula and the Maryland Bipolar were the most commonly used instruments among survey respondents, regardless of the oropharyngeal subsite.

Postoperative bleeding is one of the most common and dangerous complications of robotic oropharyngeal surgery. Stokes et al. reported a low incidence of post-TORS hemorrhage at 5.78%, with major hemorrhages requiring emergent measures like embolization, TAL, or tracheotomy in just 2.90% of cases (8). Similarly, Pollei et al., focusing exclusively on oropharyngeal TORS procedures, found a comparable bleeding rate of 5.4%, with 67.3% of these cases requiring operative intervention (9). One of the most commonly adopted methods to prevent TORS-related bleeding is the prophylactic ligation of neck vessels. Gleysteen et al. in their study found that prophylactic external carotid artery ligation did not significantly alter the overall rate of postoperative bleeding but might reduce the risk of severe, life-threatening hemorrhage (10). Despite this evidence, only 60% of TORS surgeons in our survey routinely ligate neck vessels during base of tongue or tonsil/tonsillar fossa TORS procedures. Although the overall rate of prophylactic neck vessel ligation did not differ significantly between subsites, we observed a marked difference in which vessels were chosen. Among surgeons who performed routine ligation, the lingual artery was almost universally ligated in base of tongue procedures but was ligated far less often in lateral oropharyngectomy, whereas in tonsil/tonsillar fossa TORS procedures, the facial and lingual arteries were ligated with similar frequency. This pattern may reflect differences in surgical strategy between subsites and is consistent with their differing vascular anatomy and dominant arterial supply. Lastly, among the intraoperative tools used to prevent bleeding at the end of oropharyngeal TORS procedures, hemostatic agents are commonly employed in tonsil (47.8%) and base of tongue (42%) TORS procedures, with no significant difference between subsites. The most frequently used agents include Tissuecol/Fibrin glue, Fibrillar, and Floseal.

Another preventive patient care measure commonly used to protect the airway is the placement of a temporary tracheotomy. According to our survey, this approach is primarily reserved for salvage procedures, anticipated difficult airway management, or cases involving major cervical vessel exposure. However, it is now rarely performed in all cases, with only a few centers (4-9%) opting for tracheotomy routinely, regardless of the procedure, and with no significant differences between subsites. This finding is consistent with the contemporary trend toward selective rather than routine tracheotomy after TORS. One of the recognized advantages of TORS is the possibility of avoiding tracheotomy in most patients, thereby facilitating functional recovery and reducing hospitalization. Nevertheless, postoperative hemorrhage and airway edema remain potentially life-threatening complications that may justify prophylactic airway protection in carefully selected cases (11, 12).

Current evidence suggests that tracheotomy decisions are influenced by a combination of patient-related and procedure-related factors. In a national survey of experienced French TORS teams, previous radiotherapy, anticoagulant or antiplatelet therapy, respiratory comorbidities, difficult airway characteristics, and extensive resections were among the most frequently cited indications for prophylactic tracheotomy (13). Similarly, Stringa et al. (14) recently proposed a decision-making algorithm incorporating severe obstructive sleep apnea, obesity, elevated ASA and El-Ganzouri scores, coagulopathy, cardiovascular disease, and smoking status. However, prophylactic tracheotomy is not without drawbacks. Potential complications include peristomal bleeding, dysphagia, tracheal stenosis, pneumonia, delayed swallowing rehabilitation, increased patient discomfort, and prolonged hospitalization (15). A recent systematic review by Chiari et al. (16) further demonstrated that prophylactic tracheotomy is performed in only approximately one-quarter of patients undergoing TORS for supraglottic cancer and highlighted the lack of robust evidence supporting its routine use.

Taken together, these findings suggest that airway management remains one of the least standardized aspects of perioperative TORS care. The substantial variability observed among respondents in our survey likely reflects the absence of universally accepted criteria for tracheotomy, with decisions continuing to rely largely on surgeon experience, institutional protocols, and individualized assessment of bleeding and airway risks.

Beyond airway management, postoperative functional recovery is another important aspect of perioperative care after TORS. One of the key functional advantages of robotic surgery over open surgery is the rapid resumption of a full oral diet (17, 18). No clear evidence and guidelines are available regarding the post-operative oral diet. In our survey, a nasogastric feeding tube is preventively used in 58-64% of cases of the procedures, with a significant variability measured between surgeons regarding the time to resume a full oral diet. Only a minority of surgeons aim to achieve a full oral diet in the first 3 postoperative days for base of tongue procedures (18.8%), and lateral oropharyngectomies (27.5%), and approximately one third (36.2%) of the surgeons expect to resume a full oral diet more than 1 week after the surgery. Robotic surgery appeared to be associated with shorter hospital stays compared to traditional oropharyngeal approaches in previous studies (19, 20). However, significant inter-surgeon variability was observed in our survey. Despite several cohort studies demonstrated that TORS is a safe procedure with a feasible discharge on the first postoperative day in most cases (20), the majority of surgeons reported hospitalizing patients for at least 4 days after both base of tongue procedures (76.8%) and lateral oropharyngectomies (71.0%), with no significant differences between these two subsites. Reported hospitalization durations may reflect individual surgeon practices and local approaches regarding diet resumption and postoperative monitoring, though our survey did not capture the reasons underlying these decisions or whether they were driven by formal institutional pathways.

Traditionally, TORS was associated with the treatment of smaller T1–2 tumors, with defects allowed to heal by secondary intention. However, as head and neck surgeons increasingly use robotic techniques for larger tumors, the role of robotic-assisted reconstruction has emerged. Despite this, clear guidelines on when reconstruction is necessary are lacking. The indications for using flaps, whether local or free, to reconstruct surgical defects following TORS procedures remain a topic without international consensus (21). Additionally, the effectiveness of flaps in accelerating recovery, preventing bleeding, and improving quality of life is still uncertain (22). Nearly 30% of TORS surgeons reported never using a flap following oropharyngeal TORS surgery. This finding further reflects the lack of consensus regarding reconstructive indications after TORS. However, the survey did not specifically investigate the reasons underlying the decision not to reconstruct, limiting our ability to determine whether this practice is driven by surgeon preference, case selection, institutional experience, or reliance on healing by secondary intention. Our results showed that local flap reconstruction is performed significantly more often after lateral oropharyngectomy than after base of tongue resection, a difference primarily driven by cases involving carotid artery or other major vessel exposure. The tonsillar fossa is the oropharyngeal subsite where flaps are most frequently utilized. In cases requiring reconstruction after TORS, revascularized free flaps are generally preferred over local or regional flaps, with no significant difference in free flap utilization between subsites.

While our study benefited from a broad sampling strategy and diverse participation, it has several limitations. Firstly, because TORS is practiced in a limited number of centers per country, it was not feasible to stratify the results by specific geographic areas. Although responses were distributed across the main regions where TORS is more developed, the survey primarily involved surgeons practicing in large academic and tertiary referral centers. As respondents were identified through the published TORS literature and professional networks, surgeons with greater expertise and higher procedural volumes may be overrepresented. Consequently, the findings may not fully reflect practice patterns in lower-volume or non-academic settings and should be interpreted as representative of contemporary practice among experienced TORS surgeons rather than universally generalizable to all centers performing TORS. Moreover, the responses were self-reported, and may reflect individual practices and preferences rather than formal institutional protocols, although in some cases these may overlap with center-level approaches. As participation was voluntary and the response rate was 37.9%, selection and non-response bias cannot be excluded. Surgeons with greater engagement in TORS practice, stronger opinions, or more structured institutional protocols may have been more likely to participate, potentially influencing the observed patterns of practice. Consequently, the findings should be interpreted with caution, as responders may not fully reflect the practices and perspectives of non-responders. Additionally, no specific time frame was defined for completing the survey, introducing the potential for recall bias. The nature of surveys inherently carries further limitations: the inclusion of many open-ended questions provided rich qualitative data but also introduced variability in the responses. Such variability can complicate the data analysis and interpretation processes, potentially affecting the consistency and reliability of the conclusions. Despite these limitations, the findings from this study provide valuable trends and insights into TORS practices. They highlight important considerations and areas of variability in perioperative and intraoperative patient management among experienced TORS surgeons, although they should be interpreted with caution due to the noted constraints. Importantly, the survey identified several domains where clinical practice remains heterogeneous, including bleeding prevention strategies, airway management, postoperative nutritional support, hospitalization practices, and reconstructive approaches. From a practical perspective, the findings suggest that many experienced TORS surgeons place particular emphasis on bleeding prevention, as reflected by the relatively frequent use of prophylactic vessel ligation, although its adoption was not universal and the survey design does not permit conclusions regarding its effectiveness. In contrast, the use of hemostatic agents and temporary tracheotomy demonstrated greater variability, highlighting areas where clinical decision-making remains highly individualized and where standardized recommendations are currently lacking. These areas of variability may represent priority targets for future prospective research, consensus-building initiatives, and the development of evidence-based recommendations aimed at improving consistency in perioperative TORS care.

## Conclusions

This study highlights the significant variability in perioperative and intraoperative management practices reported by experienced surgeons to reduce the risk of complications after TORS. While some aspects of care, such as the use of specific robotic instruments, appear relatively consistent, considerable heterogeneity remains regarding bleeding prevention, airway management, postoperative care, and reconstructive strategies. The indications and role of reconstruction using free or local flaps after TORS remain particularly poorly defined. These findings provide a contemporary overview of international TORS practice patterns and may help inform future research, consensus-building initiatives, and the development of evidence-based recommendations aimed at standardizing patient management and optimizing clinical outcomes.

## Data Availability

Data are available from the corresponding author upon reasonable request.
