# Supplementary material for: Preventing bleeding and surgical complications in transoral robotic surgery for the oropharynx: results of a global survey
Source: Front Oncol. 2026 Jul 20;16:1864700. doi: 10.3389/fonc.2026.1864700 (PMC13429512; doi:10.3389/fonc.2026.1864700)
Supplement: Supplementary file 1 [file Table1.docx]

**Appendix A: Survey questionnaire.**

**Table S1: McNemar’s test for paired comparison.**

**Appendix A**

The survey includes various question types, including open questions (OP), multiple-choice questions allowing for the selection of more than one answer (M), and single-choice questions (S).

1. (OP) Name and Affiliation
2. (OP) Age
3. (S) Sex
   1. Female
   2. Male
   3. Prefer not to say
   4. Other
4. (OP) Country
5. (OP) Hospital Name
6. Type of Medical Center
   1. University
   2. Non-University
7. (S) Experience in Transoral Robotic Surgery (years):
   1. <1
   2. 1-2
   3. 2-5
   4. 5-10
8. (S) Experience (average number of procedures per year as a main surgeon):
   1. 0-4
   2. 5-10
   3. 11-20
   4. 21-30
   5. 31-40
   6. 41-50
   7. >50
9. (S) Experience (average number of procedures per year as an assistant):
   1. 0-4
   2. 5-10
   3. 11-20
   4. 21-30
   5. 31-40
   6. 41-50
   7. >50
10. (S) Which robotic system do you use mainly?
    1. Da Vinci Si
    2. Da Vinci Xi
    3. Da Vinci SP
    4. Flex Medrobotics
    5. Versius CMR
    6. Other
11. (M) Name 3 types of surgical procedures you most frequently perform with the use of robotic system:
    1. Lateral oropharyngectomy/Radical tonsillectomy
    2. Base of tongue resection
    3. Supraglottic Laryngectomy
    4. Posterior pharyngeal wall resection
    5. Other
12. (M) Which of these instruments do you routinely use during base of tongue TORS procedures?
    1. Monopolar Spatula
    2. Monopolar Curved Scissors (Hot Shears)
    3. Permanent Cautery Hook
    4. Grasp Forceps
    5. Maryland Bipolar
    6. Fenestrated Bipolar Forceps
    7. Clip Applier
    8. Other
13. (M) Which of these instruments do you routinely use during tonsil/tonsillar fossa TORS procedures?
    1. Monopolar Spatula
    2. Monopolar Curved Scissors (Hot Shears)
    3. Permanent Cautery Hook
    4. Grasp Forceps
    5. Maryland Bipolar
    6. Fenestrated Bipolar Forceps
    7. Clip Applier
    8. Other
14. (S) Do you routinely ligate any vessels in the neck during base of tongue TORS procedures?
    1. Yes
    2. No
    3. Other
15. (M) If yes, which one?
    1. External carotid artery at the origin
    2. External carotid after the superior thyroid artery bifurcation
    3. Facial artery
    4. Lingual artery
    5. Ascending pharyngeal artery
    6. Other
16. (S) Do you routinely ligate any vessels in the neck during tonsil/tonsillar fossa TORS procedures?
    1. Yes
    2. No
    3. Other
17. (M) If yes, which one?
    1. External carotid artery at the origin
    2. External carotid after the superior thyroid artery bifurcation
    3. Facial artery
    4. Lingual artery
    5. Ascending pharyngeal artery
    6. Other
18. (M) When do you perform a tracheostomy during base of tongue TORS procedures?
    1. All cases
    2. Difficult intubation cases
    3. Coagulation alteration (e.g. blood thinners)
    4. Major vessels exposure
    5. Carotid exposure
    6. Intraoperative fistula
    7. Salvage surgery
    8. Other
19. (M) When do you perform a tracheostomy during tonsil/tonsillar TORS procedures?
    1. All cases
    2. Difficult intubation cases
    3. Coagulation alteration (e.g. blood thinners)
    4. Major vessels exposure
    5. Carotid exposure
    6. Intraoperative fistula
    7. Salvage surgery
    8. Other
20. (S) How long, when performed, is the tracheostomy maintained in place after base of tongue TORS procedures? (On average)
    1. 1-3 days
    2. 4-6 days
    3. 7-9 days
    4. 10-12 days
    5. >12 days
21. (S) How long, when performed, is the tracheostomy maintained in place after tonsil/tonsillar fossa TORS procedures? (On average)
    1. 1-3 days
    2. 4-6 days
    3. 7-9 days
    4. 10-12 days
    5. >12 days
22. (S) How long after base of tongue TORS procedures is the patient discharged on average?
    1. 1-3 days
    2. 4-6 days
    3. 7-9 days
    4. 10-12 days
    5. >12 days
23. (S) How long after tonsil/tonsillar fossa TORS procedures is the patient discharged on average?
    1. 1-3 days
    2. 4-6 days
    3. 7-9 days
    4. 10-12 days
    5. >12 days
24. (S) Do you routinely put a nasogastric (dobhoff) tube after base of tongue TORS procedures?
    1. Yes
    2. No
25. (S) Do you routinely put a nasogastric (dobhoff) tube after tonsil/tonsillar fossa TORS procedures?
    1. Yes
    2. No
26. (S) How long after base of tongue TORS procedures does the patient resume a full oral diet on average?
    1. 1-3 days
    2. 4-6 days
    3. 7-9 days
    4. 10-12 days
    5. >12 days
27. (S) How long after tonsil/tonsillar fossa TORS procedures does the patient resume a full oral diet on average?
    1. 1-3 days
    2. 4-6 days
    3. 7-9 days
    4. 10-12 days
    5. >12 days
28. (M) Do you usually perform flaps after oropharyngeal surgery?
    1. Never
    2. Sometimes, after base of tongue TORS procedures
    3. Sometimes, after tonsil/tonsillar fossa TORS procedures
    4. Sometimes, after soft palate TORS procedures
    5. Sometimes, after pharyngeal wall TORS procedures
29. (M) When do you perform a local flap during base of tongue TORS procedures?
    1. All cases
    2. Difficult intubation cases
    3. Coagulation alteration (e.g. blood thinners)
    4. Major vessels exposure
    5. Carotid exposure
    6. Intraoperative fistula
    7. Salvage surgery
    8. Never
    9. Other
30. (OP) Which one?
31. (M) When do you perform a local flap during tonsil/tonsillar fossa TORS procedures?
    1. All cases
    2. Difficult intubation cases
    3. Coagulation alteration (e.g. blood thinners)
    4. Major vessels exposure
    5. Carotid exposure
    6. Intraoperative fistula
    7. Salvage surgery
    8. Never
    9. Other
32. (OP) Which one?
33. (M) When do you perform a free flap during base of tongue TORS procedures?
    1. All cases
    2. Difficult intubation cases
    3. Coagulation alteration (e.g. blood thinners)
    4. Major vessels exposure
    5. Carotid exposure
    6. Intraoperative fistula
    7. Salvage surgery
    8. Never
    9. Other
34. (OP) Which one?
35. (M) When do you perform a free flap during tonsil/tonsillar fossa TORS procedures?
    1. All cases
    2. Difficult intubation cases
    3. Coagulation alteration (e.g. blood thinners)
    4. Major vessels exposure
    5. Carotid exposure
    6. Intraoperative fistula
    7. Salvage surgery
    8. Never
    9. Other
36. (OP) Which one?
37. (S) Do you routinely use hemostatic agents to prevent bleeding at the end of base of tongue TORS procedures?
    1. Yes
    2. No
38. (M) If yes, which one?
    1. Tissuecol/Fibrin Glue
    2. Floseal
    3. Integra
    4. Tabotamp
    5. Evarrest
    6. Fibrillar
    7. Other
39. (S) Do you routinely use hemostatic agents to prevent bleeding at the end of tonsil/tonsillar fossa TORS procedures?
    1. Yes
    2. No
40. (M) If yes, which one?
    1. Tissuecol/Fibrin Glue
    2. Floseal
    3. Integra
    4. Tabotamp
    5. Evarrest
    6. Fibrillar
    7. Other

**Table S1**

| **Variable** | **p-value** |
| --- | --- |
| Prophylactic neck vessel ligation | 0.84 |
| Hemostatic agent use | 0.45 |
| Routine tracheotomy | 0.50 |
| Hospitalization time | 0.39 |
| Nasogastric tube use | 0.48 |
| Local flap reconstruction | 0.009 |
| Free flap reconstruction | 0.34 |
